# The Transcriptome Trajectory Reveals Sex‐ and Age‐Dependent Changes in the Mouse Adrenal Gland

**DOI:** 10.1111/acel.70169

**Published:** 2025-07-12

**Authors:** Hui Wang, Ben Maggard, Huifei Sophia Zheng, Yuan Kang, Chen‐Che Jeff Huang

**Affiliations:** ^1^ Department of Anatomy, Physiology and Pharmacology, College of Veterinary Medicine Auburn University Auburn Alabama USA

**Keywords:** adrenal gland, age‐dependent, RNA‐seq, sex‐dependent

## Abstract

In both humans and mice, the adrenal gland is a sexually dimorphic organ, but the extent of this diversity throughout development remains unclear. Here, we analyzed the mouse adrenal gland transcriptome at postnatal days 0, 7, 15, 21, 28, 35, and 49 to uncover its transcriptomic trajectory. Sex‐dependent differences, indicated by the number of differentially expressed genes, gradually increase over time. Two Y‐linked genes are consistently expressed in male adrenal glands, suggesting that factors beyond sex hormones may contribute to adrenal sexual dimorphism. Genes involved in steroidogenesis, cholesterol synthesis, and catecholamine synthesis exhibit sex‐ and age‐dependent differential expression. Weighted gene co‐expression network analysis (WGCNA) identified many genes with known zone‐specific adrenal expression, including *Akr1c18*, *Pik3c2g*, *Cyp2f2*, *Dhcr24*, *Thrb*, and *Spp1*, clustering within the same module. FRZB, a WNT inhibitor, was also part of this module, exhibiting sex‐ and age‐dependent expression. Immunostaining confirmed that FRZB is specifically expressed in the inner cortex, aligning with other inner cortex markers. Additionally, heatmap analysis revealed that many WNT downstream genes show age‐dependent increases in expression in males, corresponding to progressively lower *Frzb* levels, suggesting a regulatory role for *Frzb* in adrenal sexual dimorphism. Furthermore, collagen‐related genes were highlighted in the clustered heatmap of all differentially expressed genes due to their gradual decrease in expression over time. These observations suggest that this comprehensive dataset not only enhances our understanding of adrenal development and sexual dimorphism, aids in identifying novel marker genes for specific adrenal cell types, but also holds potential for contributing to aging research.

## Graphical abstract

This comprehensive RNA‐seq dataset reveals the sex‐ and age‐dependent gene expression profile of the mouse adrenal gland. It helps identify novel marker genes of the adrenal inner cortex, offering new insights into the organ's development and aging process.

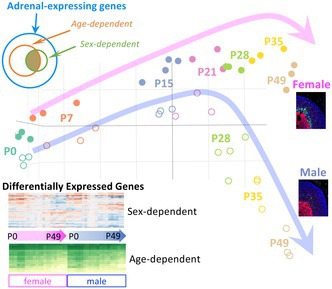

## 1 Introduction

In both humans and mice, the adrenal gland exhibits sexual dimorphism across various levels, including histology, gene expression, and function (Kudielka and Kirschbaum 2005; Kloehn et al. 2016). Many adrenal diseases also show a strong sex bias (Bechmann et al. 2024). In mice, the sexual dimorphism in the adrenal gland has been noticed for more than 90 years as the innermost layer of the adrenal cortex (X‐zone) regresses by puberty in males and during the first pregnancy in females (Kang et al. 2024). A transcriptomic study using human adrenal samples also reveals sex‐related diversity in human adrenocortical cells at single‐cell resolution (Huang et al. 2021). In mice, sex differences in the adrenal gland have been observed at the histological, hormonal, and gene expression levels using various models, including mice of different ages, with and without gonadectomy, and mice treated with different hormones (Spinedi et al. 1992; Bielohuby et al. 2007; El Wakil et al. 2013; Lyu, Wang, et al. 2020; Ruggiero et al. 2021). However, little is known about how this sexual dimorphism evolves throughout the prepubertal and pubertal developmental process. Although many transcriptomic studies provide a comprehensive view of the human and mouse adrenal glands, either using bulk RNA‐seq or single‐cell resolution, the age‐dependent transcriptome profiles of the adrenal gland remain unclear. In this study, we use bulk RNA‐seq to compare gene expression profiles in mouse adrenal glands at postnatal days (P) 0, 7, 15, 21, 28, 35, and 49 in both sexes. These data uncover the age‐ and sex‐dependent transcriptome trajectories of the mouse adrenal gland and serve as a fundamental reference resource for adrenal gland research.

## 2 Material and Method

### 2.1 Animals

C57BL/6 breeding pairs were housed under a 12:12‐h light/dark cycle with free access to water and standard rodent chow. Newborn mice were designated as P0 on the day of birth. Mice at P7 were euthanized by decapitation, while mice aged P15 and older were euthanized by CO_2_ inhalation followed by decapitation. Mice were euthanized in the afternoon, between 2 and 4 h before the onset of the dark cycle (lights off at 6:00 PM). Adrenal glands were immediately collected post‐euthanasia, and surrounding adipose tissue was carefully removed. Collected adrenal glands were snap‐frozen in liquid nitrogen and stored at −80°C until the RNA extraction. All procedures were conducted in accordance with protocols approved by the Institutional Animal Care and Use Committee (IACUC) at Auburn University.

### 2.2 RNA Isolation and Sequencing

Total RNA was extracted from frozen adrenal glands using the Monarch Total RNA Miniprep Kit (New England Biolabs, Ipswich, MA) following the manufacturer's user manual. For each biological replicate (i.e., RNAseq library), adrenal glands from 2 to 3 animals were pooled for total RNA extraction, with 3–4 libraries for each time point for each sex. Total RNA samples were sent to Novogene Life Sciences Co. Ltd. for paired‐end 150 bp RNA sequencing (PE150 RNA‐seq). Only samples that passed quality control (concentration > 25 ng/μL, RIN ≥ 7.5) were used for library preparation and subsequent RNA sequencing. To obtain gene‐specific read counts, unstranded paired‐end read files were initially analyzed using the FASTQ error check feature in Chipster (Kallio et al. 2011). Quality control was then performed on the raw read files using FastQC. The trimmed reads were aligned to the mouse genome (mm10, GRCm38) using STAR (v. 2.5) (Dobin et al. 2013) with the Maximal Mappable Prefix (MMP) method ensuring precise mapping of exon‐exon junction reads. HTSeq (v.0.6.1) (Anders et al. 2015) was used to generate the final read counts mapped of each gene.

### 2.3 Bioinformatics Data Analysis

Differential expression analysis between groups was conducted using DESeq2 (Love et al. 2014) in R version 4.3.2, with a false discovery rate (FDR) cutoff of *α* = 0.05. Statistical significance of differential expression was assigned to genes with adjusted *p*‐values < 0.05 and a fold change > 1.5. Statistically significant genes were further classified as differentially expressed genes (DEGs) if they exhibited at least five fragments per kilobase per million mapped reads (FPKM) in at least one sample. Heatmaps of DEGs were generated using the heatmap.2 function in R, applying complete linkage clustering with Euclidean distance measurement under default settings (Figures 2 and 6) or Ward. D2 linkage clustering with Manhattan distance measurement (Figure 4). Log2 fold change for the heatmap was calculated by adding 0.001 to each FPKM value and comparing it to the average FPKM value at P21 for each sex. Gene Ontology (GO) analysis was performed using the enrichGO function in the clusterProfiler package (version 3.16.0) and the Panther Gene Ontology website (geneontology.org). GO‐Figure! was also used for visualization as shown in Figure 2 (Yu et al. 2012; Raudvere et al. 2019). Weighted correlation network analysis (WGCNA) was performed using the WGCNA package in R (Langfelder and Horvath 2008). Genes with counts of 15 or higher in more than 65% of the samples were included in the analysis, and a soft‐thresholding power of 12 was applied. The over‐representation of WGCNA modules in each functional category shown in Figure 6 was assessed using a hypergeometric test. The test evaluated whether the number of genes shared between a module and a functional category was significantly higher than expected by chance, given the total number of genes in the dataset. A *p*‐value was computed for each module‐category pair, and a significance threshold of *p* < 0.05 was applied to identify enriched modules.

### 2.4 Immunostaining

Tissues were fixed in 4% paraformaldehyde in phosphate‐buffered saline on ice for at least 6 h. They were then processed through standard paraffin embedding and sectioning procedures. Sections were dewaxed, rehydrated, antigen retrieved, and blocked, followed by the application of primary and secondary antibodies as previously described (Lyu, Zheng, et al. 2020). The following antibodies were used: rabbit anti‐FRZB primary antibody (1:500 dilution, PA5104120, ThermoFisher), mouse anti‐tyrosine hydroxylase primary antibody (1:1000 dilution, sc‐25269, Santa Cruz), donkey anti‐rabbit biotinylated secondary antibody (1:500 dilution, 711‐066‐152, JacksonImmuno), and donkey anti‐mouse Cy3 secondary antibody (715‐166‐151, JacksonImmuno). The signal amplification was performed using fluorescein tyramide for FRZB, and DAPI (4′,6‐diamidino‐2‐phenylindole) was used to stain cell nuclei. Adrenal glands from at least three mice were examined for each sex and age group.

## 3 Results

**FIGURE 1:**
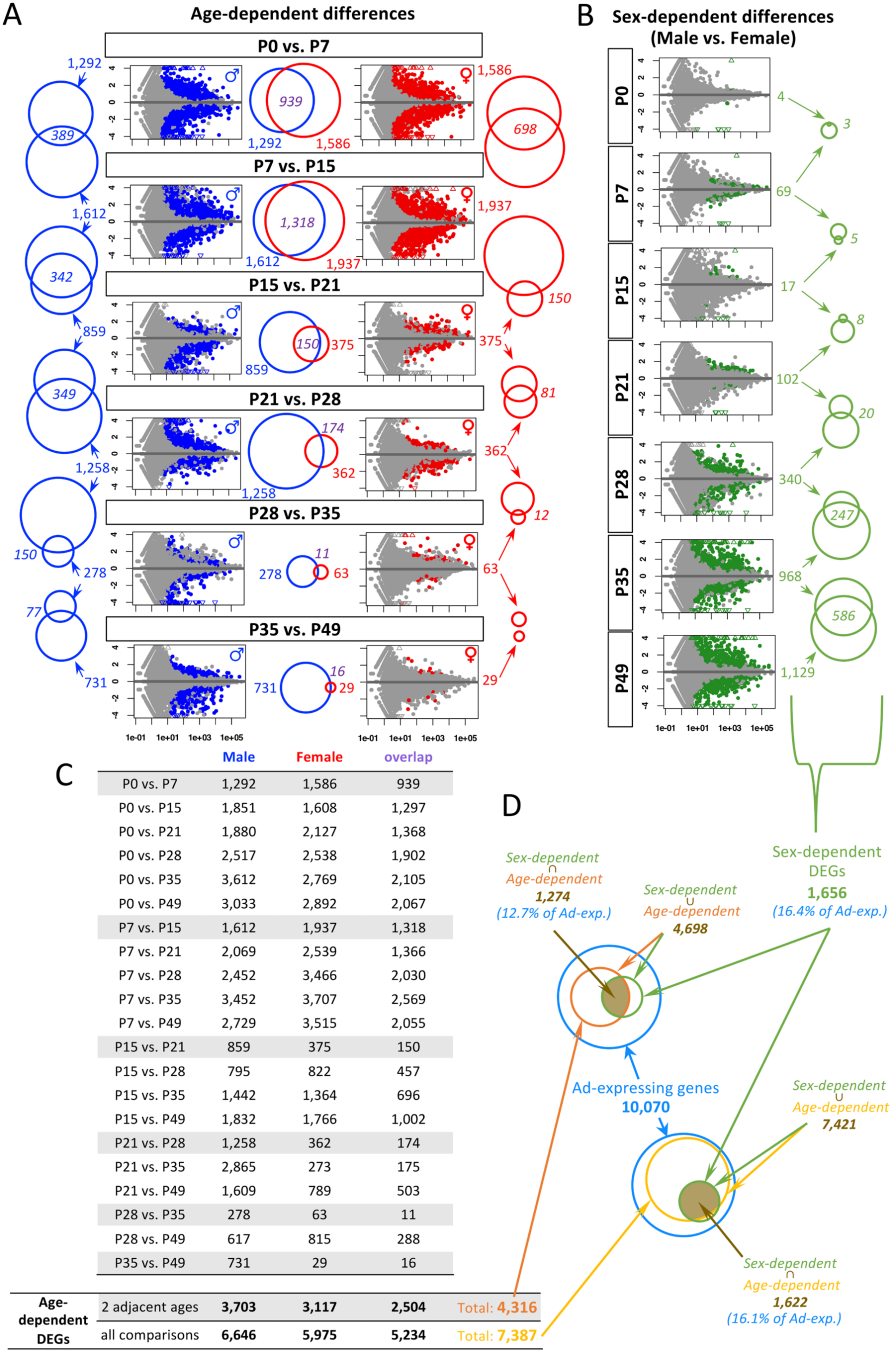
Differentially expressed genes (DEGs) were identified using DESeq2, incorporating all replicates in each group. (A and B) MA plots provide an overview of differential gene expression in each comparison, while Venn diagrams compare the numbers of DEGs across different comparisons. (A) For age‐dependent differences, DEGs were identified between adjacent ages within the same sex. (B) For sex‐dependent differences, DEGs were determined by comparing males and females within the same age group. (C) Age‐dependent DEGs can also be identified across any two different ages, increasing the total number of DEGs from 4316 (between adjacent ages) to 738 (across any two ages). (D) Venn diagrams show the overlap between sex‐dependent DEGs and age‐dependent DEGs. Ad‐expressing genes are defined as those with an FPKM of at least 5 in at least one of the 44 total samples.

The study starts with identifying the differentially expressed genes (DEGs) in the adrenal glands across varying ages and sexes. To explore how gene expression changes over time and between sexes, we performed a comprehensive analysis comparing gene expression patterns in both sexes at different ages. DEGs were identified by comparing data across different ages within the same sex (age‐dependent differences, Figure 1A) and between sexes at the same age (sex‐dependent differences, Figure 1B). Venn diagrams illustrate how these DEGs evolve over time, showing changes in the number of DEGs and the overlap between sex and age groups.

For the age‐dependent differences between two adjacent ages (Figure 1A), both males and females showed that the P0 versus P7 and the P7 versus P15 comparisons yielded the top two highest numbers of DEGs, with a significant overlap of 939 and 1318 genes between sexes. The number of overlapping DEGs decreased approximately tenfold every 2 weeks over time. For the P15 versus P21 and P21 versus P28 comparisons, the number of overlapping DEGs dropped sharply to 150 and 174, respectively. Moreover, for the P28 versus P35 and P35 versus P49 comparisons, the number further dropped to 11 and 16, respectively. This pattern corresponds with the trend observed in sex‐dependent differences (Figure 1B), as male and female transcriptomes are initially very similar from P0 to P15, with an increasing number of DEGs between sexes throughout development starting from P21 (102 at P21; 340 at P28; 968 at P35, and 1129 at P49). Hence, as development progresses, the overlap in age‐dependent DEGs between sexes decreases, and sex‐specific differences become more pronounced, reflected in the increasing number of sex‐dependent DEGs at later time points.

Particularly in females, the decreasing number of age‐dependent DEGs every 2 weeks over time (1586 and 1937 for P0 vs. P7d and P7 vs. P15; 375 for P15 vs. P21; 362 for P21 vs. P28; 63 for P28 vs. P35; and 29 for P35 vs. P49) suggests that developmental events in the female adrenal gland are primarily concentrated during the neonatal stages, prior to weaning. In males, while reports indicate X‐zone regression between P28 and P35 (Kang et al. 2024) and histological analyses reveal no significant structural differences in the adrenal gland between P35 and P49 (Huang et al. 2015), a marked increase in DEGs in males between P28 versus P35 and P35 versus P49 (2.6 times more, with 278 vs. 731 DEGs, respectively) suggests that the male adrenal gland continues to undergo developmental changes post‐weaning, leading to substantial alterations at the gene expression level.

For age‐dependent differences, comparisons can be made between two adjacent ages (gray rows in Figure 1C) or any two distinct ages (white rows in Figure 1C), resulting in a total of 4316 DEGs and 7387 DEGs, respectively. Notably, these 7387 age‐dependent DEGs include 97.9% of the sex‐dependent DEGs (1622 out of 1656; Figure 1D). This overlap suggests that the majority of sex‐dependent DEGs are also age‐dependent, implying that developmental processes contribute significantly to the sexual dimorphism of the adrenal gland. Among all adrenal‐expressing genes (defined as genes with at least 5 FPKM in at least one sample at any age from P0 to P49), only 16.4% are sex‐dependent DEGs. Consequently, only 12.7% to 16.1% of adrenal‐expressing genes are both sex‐ and age‐dependent (Figure 1D).

Notably, when comparing chromosomal distribution between sex chromosomes and autosomes, two Y‐linked genes are consistently identified as differentially expressed across all sex‐dependent comparisons (Table 1). While both genes are exclusively expressed in males at all ages, their expression levels vary across different ages (FPKM ranging from 9.9 to 23.9 for *Eif2s3y* and 13.5 to 42.0 for *Ddx3y*). These fluctuations in expression also lead to their identification as age‐dependent DEGs in some comparisons between adjacent age groups.

**TABLE 1:** Distribution of DEGs across X, Y, and autosomal chromosomes.

	Number of DEGs		
	Autosomal	X‐linked	Y‐linked (*gene names*)
Age‐dependent			
Male			
P0 vs. P7	1234	56	2 (*Eif2s3y, Gm47283*)
P7 vs. P15	1561	51	0
P15 vs. P21	821	37	1 (*Ddx3y*)
P21 vs. P28	1206	51	1 (*Ddx3y*)
P28 vs. P35	267	11	0
P35 vs. P49	699	31	1 (*Ddx3y*)
Female			
P0 vs. P7	1528	58	0
P7 vs. P15	1871	66	0
P15 vs. P21	355	20	0
P21 vs. P28	354	8	0
P28 vs. P35	62	0	1 (*Gm47283*)
P35 vs. P49	29	0	0
Sex‐dependent			
P0	2	1	2 (*Ddx3y, Eif2s3y*)
P7	59	8	2 (*Ddx3y, Eif2s3y*)
P15	13	2	2 (*Ddx3y, Eif2s3y*)
P21	97	3	2 (*Ddx3y, Eif2s3y*)
P28	321	17	2 (*Ddx3y, Eif2s3y*)
P35	920	45	3 (*Ddx3y, Eif2s3y, Gm47283*)
P49	1073	53	3 (*Ddx3y, Eif2s3y, Gm47283*)

*Note:* Although Gm47283 is listed as a Y‐linked gene, its paralog Gm21887 is located on the X chromosome. Gm47283 is expressed in female samples in our dataset.

**FIGURE 2:**
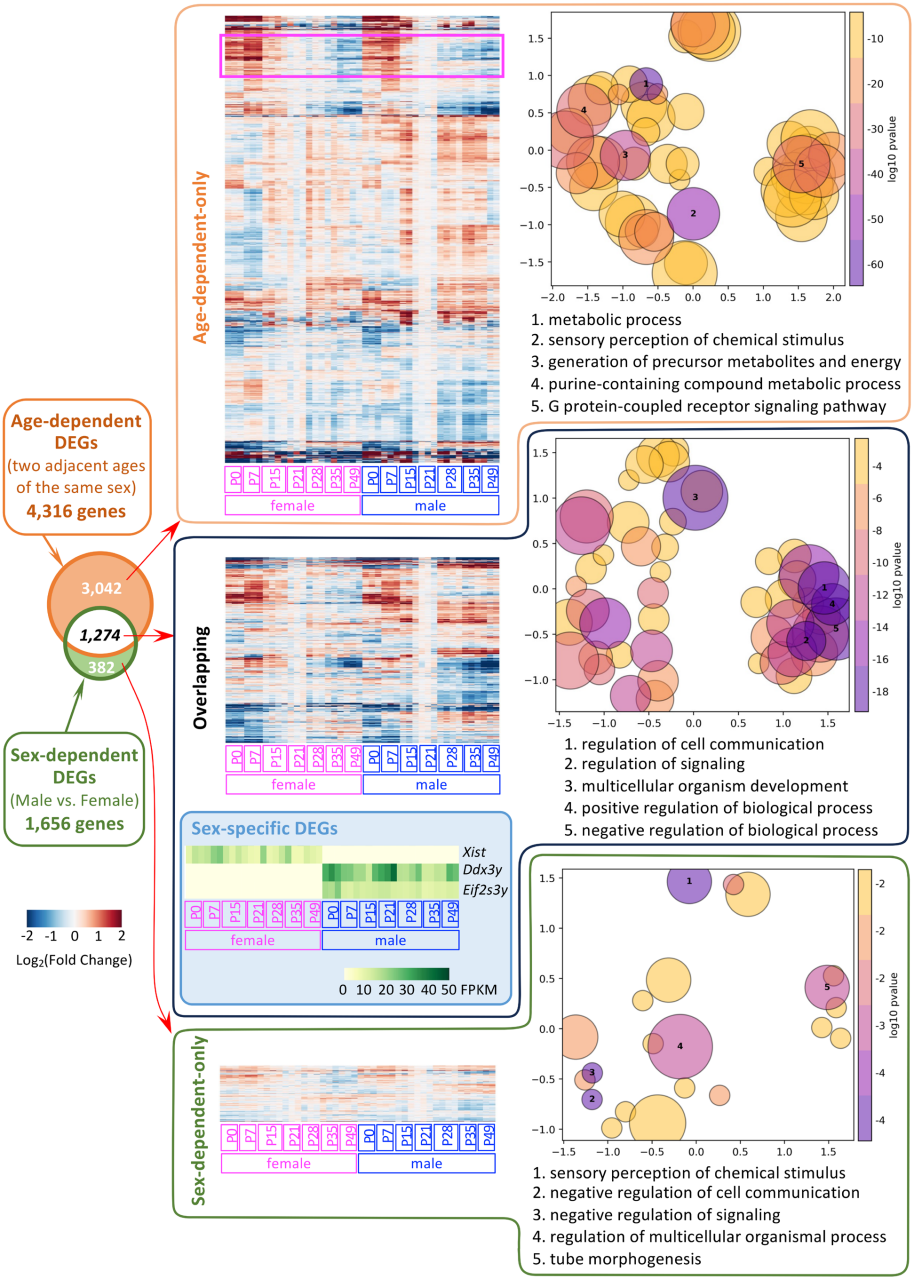
A Venn diagram compares the age‐dependent DEGs with the sex‐dependent DEGs. The heatmap illustrates the expression patterns of DEGs in an age‐dependent manner. For each sex, gene expression at P21 is used as the baseline to calculate fold changes. GO analysis highlights the top GO terms for age‐dependent‐only DEGs, sex‐dependent‐only DEGs, and DEGs influenced by both sex and age.

To reveal the age‐dependent and sex‐dependent patterns of gene expression, we used clustered heatmaps to display the fold change of DEGs between two adjacent ages, sex‐dependent DEGs, and the overlapping DEGs (Figure 2). The heatmap reveals a clear age‐dependent pattern in both the age‐dependent‐only and overlapping categories. Even in the sex‐dependent‐only group, an age‐dependent pattern is still evident. More interestingly, although *Ddx3y* and *Eif2s3y* are exclusively expressed in male samples, and *Xist* is exclusively expressed in female samples, all three genes are identified as sex‐ and age‐dependent DEGs and are in the overlapping category.

When comparing the expression patterns of DEGs across the three categories, it is evident that age‐dependent‐only DEGs exhibit substantial fold changes over time even at the early postnatal stages. For example, genes highlighted in the pink box within the age‐dependent‐only group show high expression at P0 and P7, with levels gradually declining from P15 to P49 in both sexes. In contrast, DEGs classified solely as sex‐dependent generally display weaker age‐associated trends, with smaller fold changes compared to the other two categories. For those sex‐dependent‐only DEGs, the major sex‐dependent differences emerge at later time points, particularly at P35 and P49. This pattern is consistent with the data shown in Figure 1, where the number of sex‐dependent DEGs is low during early postnatal stages, while a large number of age‐dependent DEGs are observed at these earlier time points. Together, these observations support the idea that, in the adrenal gland, age‐dependent transcriptional changes lay the foundation for sexual divergence, which is further driven toward sexual dimorphism by the androgen surge during puberty. Interestingly, Gene Ontology (GO) analysis reveals that DEGs identified solely as age‐dependent are associated with metabolism‐related processes, suggesting that adrenal gland function is still developing or undergoing changes during postnatal stages. This occurs despite the fact that all steroidogenic enzymes are expressed as early as the embryonic stage and that the adrenal gland attains steroidogenic functionality with physiological significance by the prenatal stage (Huang et al. 2012).

**FIGURE 3:**
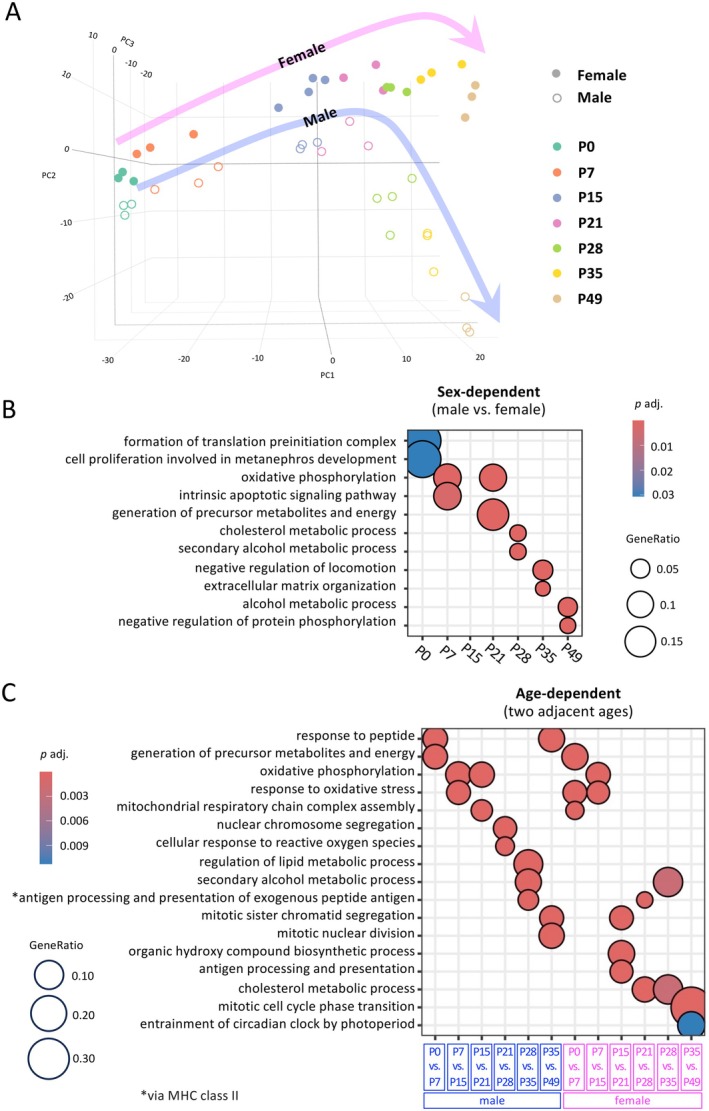
(A) Replicates are clustered in the PCA plot, revealing age‐dependent trajectories of the mouse adrenal gland transcriptome of each sex. Male samples begin to diverge from female samples starting at P28, leading to a sexually dimorphic trajectory. (B and C) GO analysis highlights the top GO terms associated with sex‐dependent (B) and age‐dependent DEGs (C). GO enrichment analysis was conducted using the enrichGO function in clusterProfiler, with redundancy reduced by applying the simplify function at a cutoff of 0.3 (using adjusted *p*‐values) to reduce term redundancy.

Using three‐dimensional Principal Component Analysis (PCA), RNA‐seq replicates cluster distinctly by developmental stages and sexes, revealing an age‐dependent trajectory in both males and females with clear sexual dimorphism (Figure 3A). From P0 to P21, male data points lie close to female data points along a similar trajectory. However, starting at P28, the male trajectory begins to diverge from the female trajectory. GO analysis of sex‐ and age‐dependent DEGs reveals that these genes are associated with distinct biological processes at different developmental stages (Figure 3B,C). Consistent with the high overlap of age‐dependent DEGs shown in Figure 1A, males and females share similarities in GO terms among age‐dependent DEGs, particularly during early developmental stages such as the P0 versus P7 and P7 versus P15 comparisons (Figure 3C). However, at later stages, females begin to exhibit unique GO terms specific to their sex. For example, the terms “generation of precursor metabolites and energy” and “response to oxidative stress” are identified in both sexes during the P0 versus P7 and P7 versus P15 comparisons, respectively. In contrast, the term “mitotic cell cycle phase transition” is exclusively observed in females during the P35 versus P49 comparison.

**FIGURE 4:**
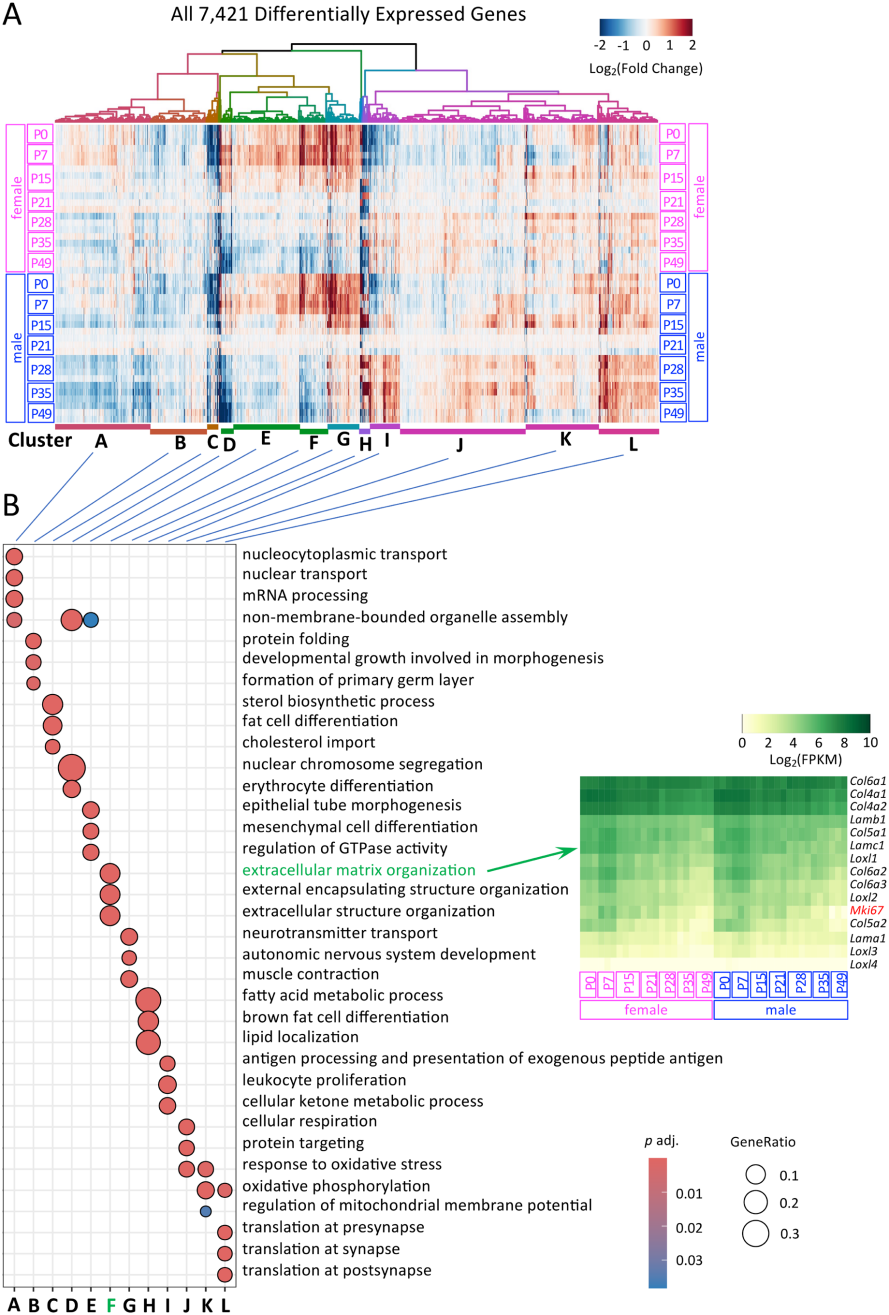
(A) Clustered heatmap of all DEGs. The 7421 DEGs include (1) 1656 sex‐dependent DEGs and (2) 7387 age‐dependent DEGs, identified from comparisons between any two age groups, including both adjacent and non‐adjacent age groups. Fold changes were calculated relative to the average expression of the P21 group within the same sex. (B) GO enrichment analysis was conducted using the enrichGO function in clusterProfiler, with redundancy reduced by applying the simplify function at a cutoff of 0.3 (using adjusted *p*‐values).

The clustered heatmap of all 7421 DEGs, comprising both sex‐dependent and age‐dependent DEGs (as illustrated in the lower Venn diagram in Figure 1D), reveals distinct clusters exhibiting clear sex‐ and/or age‐dependent expression patterns (Figure 4A). For example, the expression levels of genes in Clusters E and F decrease over time in both sexes, whereas Clusters A and G display sexually dimorphic expression patterns at specific developmental stages. GO analysis further indicates that each cluster is associated with distinct biological processes (Figure 4B). The hierarchical clustering of all DEGs groups functionally related genes based on their expression similarities, as genes involved in similar biological processes often exhibit similar expression patterns (Eisen et al. 1998). This comprehensive dataset enables the visualization of how gene expression within a functional group changes in an age‐ and/or sex‐dependent manner. For example, “extracellular matrix organization” is identified as one of the GO terms in Cluster F. The expression of key extracellular matrix components, including collagens, laminins, and remodeling enzymes, progressively decreases over time. This is consistent with reduced cell proliferation during aging, as indicated by declining levels of the proliferation marker gene *Mki67* (Figure 4B).

**FIGURE 5:**
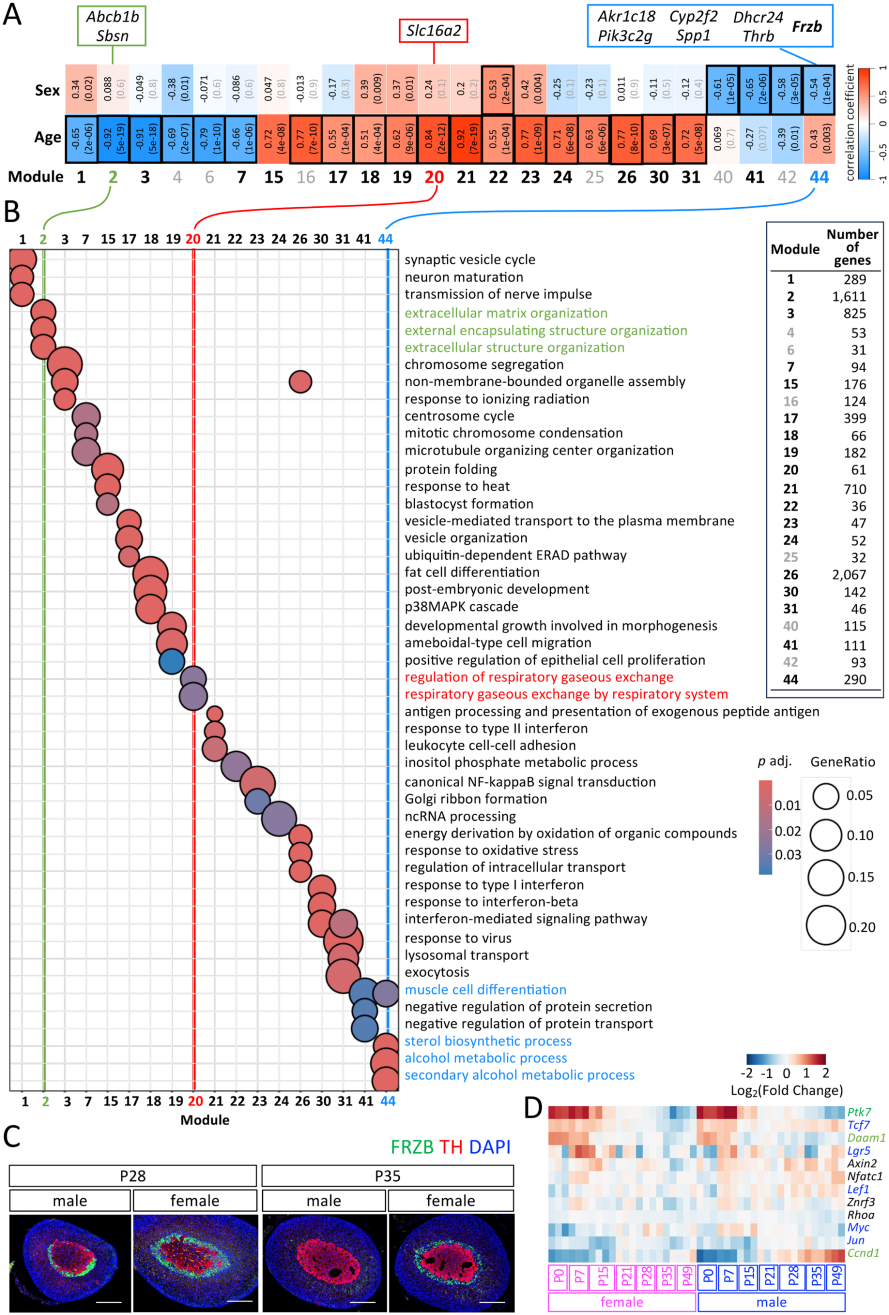
(A) WGCNA identified 44 gene modules based on correlations with age and sex. Of these, 24 modules, which had at least one correlation coefficient either > 0.5 or < −0.5, are shown in the figure. Each module has two boxes that display two correlation coefficients (one for age and one for sex), with *p*‐values provided in parentheses. Genes with distinct inner cortical expression patterns were distributed across three modules, the majority of them grouped in Module 44. (B) GO enrichment analysis was performed using the enrichGO function in clusterProfiler, with the simplify function applied at a cutoff of 0.3 (using adjusted *p*‐values). The number of genes in each module is shown. Modules with the module numbers labeled in gray had no identified GO terms. (C) Double immunostaining shows the FRZB expression in the adrenal gland at P28 and P35 in both sexes. Scale bars: 200 μm. (D) Clustered heatmap of some downstream genes of the WNT signaling pathway. Genes identified as age‐dependent (any two distinct ages) DEGs are labeled in green, and those identified as both sex‐ and age‐dependent DEGs are labeled in blue.

To further explore gene co‐expression patterns and identify functionally related gene modules, we applied Weighted Gene Co‐expression Network Analysis (WGCNA) to our dataset. WGCNA identified 44 gene modules, of which 24 exhibited distinct correlation patterns (correlation > 0.5 or < −0.5) with either age or sex (Figure 5A). Most modules correlated individually with either age or sex, except Module 22, which was influenced by both factors. Module 44, notably, showed a negative correlation with sex and clustered six previously reported inner cortex marker genes including *Akr1c18* (Hershkovitz et al. 2007), *Pik3c2g* (Pihlajoki et al. 2013), *Cpy2f2* (Lyu, Wang, et al. 2020), *Spp1* (Lyu, Wang, et al. 2020), *Dchr24* (Lyu, Wang, et al. 2020), and *Thrb* (Huang et al. 2015), while the other three inner cortex marker genes including *Abcb1b* (Lopez et al. 2021), *Sbsn* (Lopez et al. 2021), and *Slc16a2* (Cleary et al. 2019) were distributed across two additional modules.

Similar to the clustered heatmap, which groups genes using different approaches compared to WGCNA, the GO analysis of genes in each module also shows that different modules are associated with distinct GO terms (Figure 5B). It is important to note that some modules, despite containing a substantial number of genes and showing clear correlations with age or sex, still lack associated GO terms (e.g., Module 16).

The clustering of inner cortex marker genes in Module 44 prompted us to investigate whether other genes in this module might also be specific to the inner cortex. It is interesting to note that among the highly expressed genes in Module 44 exhibiting a temporal expression pattern similar to *Akr1c18*, *Frzb* ranked third when sorted by fold change between P21 and P49 males (*Akr1c18* ranked first, *Cyp2f2* second, and *Spp1* fourth). In addition, *Frzb* consistently shows sexually dimorphic expression across multiple omics studies (El Wakil et al. 2013; Dumontet et al. 2018; Lyu, Wang, et al. 2020), and its expression is modulated by gonadectomy and androgen treatment—factors linked to the presence of the adrenal gland X‐zone (El Wakil et al. 2013; Dumontet et al. 2018). Based on its strong expression at P21 and dynamic regulation, we selected FRZB for immunostaining to examine the spatial distribution of FRZB. Our results revealed that FRZB could also serve as an inner cortex marker gene in the mouse adrenal gland (Figure 5C). Since FRZB is a WNT inhibitor, it is interesting to note that the heatmap of certain WNT downstream genes shows a “warmer” pattern in males (Figure 5D), corresponding to the downregulation of *Frzb* beginning at P28, suggesting a potential loss of WNT inhibition due to the decrease in *Frzb* starting at P28.

**FIGURE 6:**
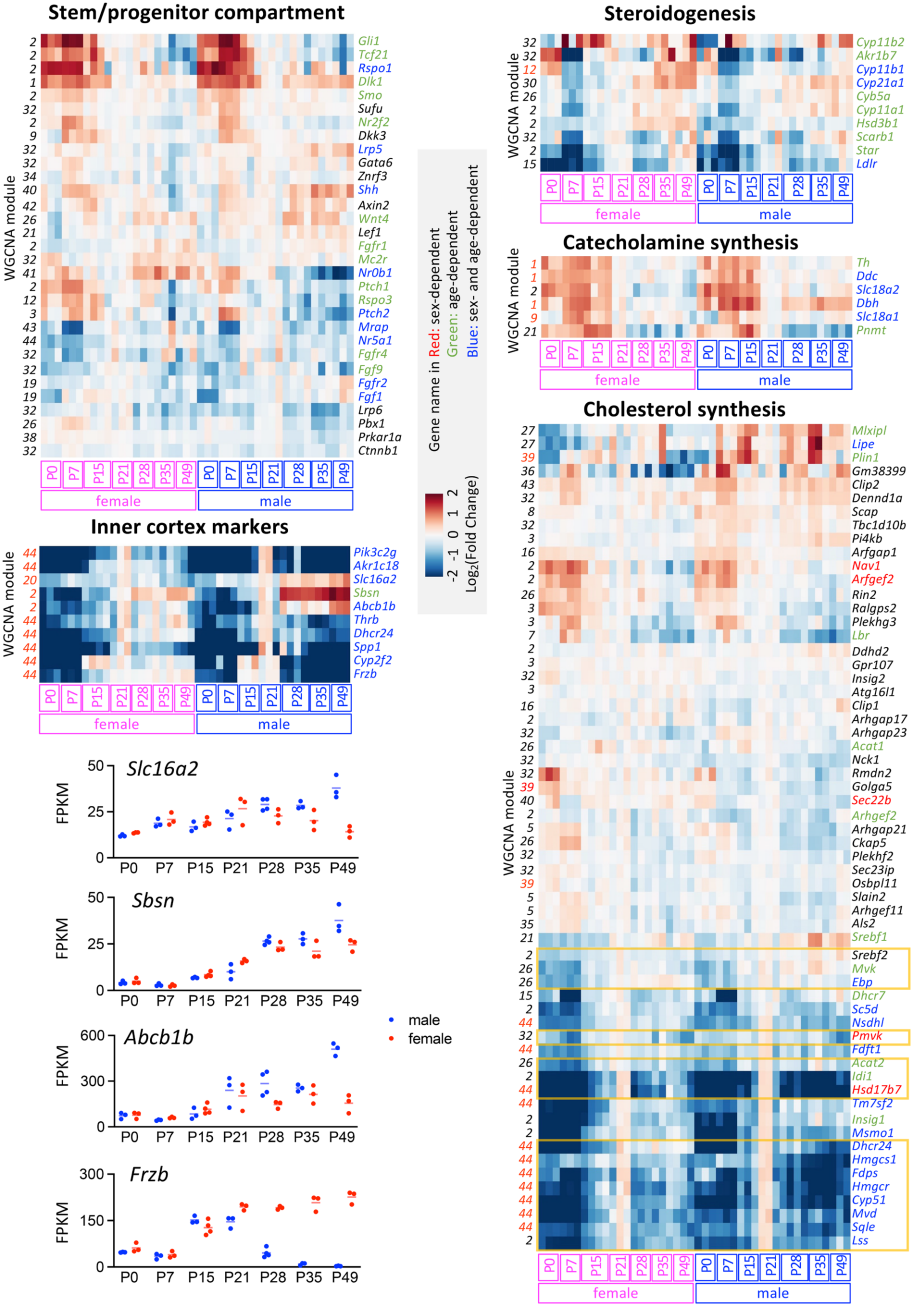
Clustered heatmap of genes categorized by specific functional groups. Genes identified as sex‐dependent DEGs are labeled in red, age‐dependent DEGs (any two distinct ages) in green, and those identified as both sex‐ and age‐dependent DEGs in blue. The numbers on the left side of the heatmap indicate the WGCNA module associated with each gene, with over‐represented modules (*p* < 0.01) highlighted in red. In the cholesterol synthesis heatmap, key enzymes and regulators of the mevalonate pathway are boxed in yellow rectangles. Among the inner cortex markers, *Slc16a2*, *Abcb1b*, and *Sbsn* exhibit a unique sex‐dependent pattern on the heatmap, with males showing increased expression levels starting from P28, in contrast to markers like *Frzb*, which display decreased expression from P28. The FPKM expression levels of these four genes are presented in dot plots.

For various gene sets related to adrenal gland function and development (Figure 6), the clustered heatmaps revealed an overall age‐dependent expression pattern, with some genes within each set also showing significant sex‐dependent differences. For example, among the 31 genes identified as key stem/progenitor cell markers, 21 display an age‐dependent pattern. Among these 21 genes, 9 of them are also identified as sex‐dependent DEGs. Specifically, *Gli1* and *Tcf21*, which are expressed in the adrenal capsule (Huang et al. 2010; Wood et al. 2013), show a progressive downregulation from P0 to P49. In contrast, *Nr0b1*, whose loss‐of‐function mutations cause congenital adrenal hypoplasia (Muscatelli et al. 1994; Zanaria et al. 1994), is upregulated in females after P21 but downregulated in males after P21. This pattern is consistent with previous findings at both mRNA and protein levels (Mukai et al. 2002).

For inner cortex marker genes, all but *Sbsn* were identified as both sex‐ and age‐dependent DEGs. *Sbsn* was age‐dependent only, with upregulation observed in both males and females starting from P28. Most inner cortex marker genes exhibit decreased expression in males starting from P28, corresponding to the regression of the 20αHSD‐positive X‐zone. However, *Slc16a2*, *Abcb1b*, and *Sbsn* display an opposite pattern, with their expression upregulated in males starting from P28.

Thyroid hormone has been reported to exert sexually dimorphic effects on the adrenal gland, with treatment inducing dramatic histological changes primarily in the inner cortical region (Lyu, Wang, et al. 2020). This zonal‐specific thyroid hormone‐mediated effect corresponds to the zonal‐specific expression of *Thrb* (encoding thyroid hormone receptor β) and *Slc16a2* (encoding the thyroid hormone transporter MCT8), which are identified as marker genes of the inner cortex (Huang et al. 2015; Cleary et al. 2019). Although both genes are also classified as sex‐ and age‐dependent DEGs in this dataset (Figure 6), they are assigned to different modules. *Thrb* is found in a sex‐correlated module, whereas *Slc16a2* is assigned to an age‐correlated module. Their expression patterns on the heatmap also differ significantly: *Slc16a2* shows increased expression in males at P28, P35, and P49, similar to *Sbsn* and *Abcb1b*, a pattern distinct from *Thrb* and most other inner cortex marker genes.

Notably, all genes involved in steroidogenesis exhibit age‐dependent expression patterns, with some also showing sex‐dependent variations. In both males and females, many genes encoding steroidogenic enzymes are upregulated starting from P28. This is particularly striking in females: especially the overall number of age‐dependent DEGs in females substantially decreases after P21 (as shown in Figure 1C, with only 63 DEGs between P28 and P35 and just 29 DEGs between P35 and P49). This highlights the unique temporal regulation of steroidogenic pathways during the transition from pre‐puberty to puberty. Similarly, all genes associated with catecholamine synthesis are identified as age‐dependent DEGs, with 4 out of 6 also displaying sex‐dependent differences. However, in contrast to steroidogenic genes, catecholamine synthesis genes generally show decreased expression at later postnatal stages, compared to higher expression levels between P0 and P15. Although *Pnmt* is only identified as an age‐dependent DEG, likely due to high variation among replicates in female samples, its expression, as shown on the heatmap, significantly decreases from P28 onward, especially in males. Overall, these expression patterns of key enzymes and regulatory factors in steroidogenesis and catecholamine synthesis highlight the distinct maturation processes of the adrenal cortex and medulla from pre‐puberty to puberty.

Genes associated with cholesterol synthesis display a unique clustering pattern on the heatmap, with genes in the mevalonate pathway grouping together. Most of these genes exhibit a peak expression pattern, characterized by lower expression levels before and after P21, with the highest expression observed at P21 in both sexes.

WGCNA operates under the expectation that genes with similar functions tend to exhibit correlated expression patterns and, consequently, cluster into the same module. In our analysis, certain WGCNA modules were significantly enriched in specific functional categories (Figure 6). For example, within the cholesterol synthesis category, statistical expectations predicted that 1.2 of the 60 genes would belong to Module 44; however, 11 genes were identified in this module (*p* < 0.001). Similarly, in the catecholamine synthesis category, three out of six genes were found in Module 1 (*p* < 0.001). Interestingly, in the stem/progenitor compartment category, Module 2 is overrepresented with a *p*‐value of 0.0504. Although the *p*‐value is not within the threshold we set, GO analysis of genes in Module 2 still identifies several GO terms related to structural organization, development, and morphogenesis.

## 4 Discussion

This comprehensive omics dataset of the mouse adrenal gland from postnatal days 0 to 49 reveals age‐ and sex‐dependent dynamic changes. Genes involved in steroidogenesis, cholesterol metabolism, stem/progenitor cell‐related pathways, and catecholamine synthesis exhibit distinct age‐ and sex‐specific expression patterns. These results highlight the developmental trajectory of the adrenal gland transcriptome and suggest that age‐dependent differences are not solely attributable to the formation and regression of the X‐zone cells.

Although the presence of the X‐zone is regulated by sex hormones, the sexual dimorphism observed in our dataset at neonatal stages before puberty suggests that adrenal gland sex differences could be governed by mechanisms beyond hormonal control. *Ddx3y* and *Eif2s3y* are Y‐linked genes expressed exclusively in male tissues, and their expression is not directly regulated by sex hormones. For example, *Eif2s3y* is highly expressed in the male mouse brain before gonadal differentiation (around embryonic Days 12–14) and persists into adulthood independently of gonadal hormone secretion (Zhang et al. 2021). Similarly, *Ddx3y* shows consistent expression in male tissues regardless of the animal's gonadal sex (Ocañas et al. 2022). The sexually dimorphic expression of *Ddx3y* and *Eif2s3y* across all analyzed ages suggests that sex hormones may not be the sole factors that make the adrenal gland sexually dimorphic. However, the expression of *Ddx3y* and *Eif2s3y* does not necessarily mean that they have a function in the adrenal gland. Whether these Y‐linked genes directly contribute to broader sex‐specific gene expression patterns in the adrenal gland remains to be determined in future studies.

Among the reported inner cortex markers, *Slc16a1*, *Abcb1b*, and *Sbsn* cluster together on the heatmap due to their upregulation in males starting at P28, a pattern that differs significantly from other inner cortex markers. This finding aligns with previous observations that the adrenal gland's inner cortex comprises distinct subzones characterized by specific marker genes. The distribution of inner cortex marker genes across different WGCNA modules further suggests that each adrenocortical subzone within the inner cortex may be regulated by distinct age‐ and sex‐dependent mechanisms. Interestingly, *Sbsn* and *Abcb1b* are not only clustered next to each other on the heatmap but also belong to the same WGCNA module. Both genes are reported as inner cortex markers that are upregulated under stress (Lopez et al. 2021). This finding highlights the effectiveness of using WGCNA in this dataset to identify genes with similar expression patterns, providing an opportunity to further group genes based on their interaction and function.

A similar divergence is observed in *Slc16a2* and *Thrb*, the two genes associated with thyroid hormone signaling and expressed in the inner cortex. Interestingly, another thyroid hormone receptor, *Thra*, which is highly expressed in the adrenal gland, is characterized in another age‐associated WGCNA module (Module 17). The distribution of the two thyroid hormone receptors and the transporter in age‐associated and sex‐associated modules suggests that thyroid hormone signaling in the adrenal gland may also exhibit age‐dependent effects, similar to its role in other organs, such as the retina and cochlea (Ng et al. 2015; Henning and Szafranski 2016). Further studies will be needed to elucidate the potential age‐dependent effects of thyroid hormone in the adrenal gland.

Immunostaining results showing FRZB as a marker for the inner cortex in mice are particularly intriguing, as FRZB has previously been reported predominantly in the zona fasciculata (zF) in rats (Nishimoto et al. 2012). A key difference between the two species is the structural composition of the inner cortex: in rats, the inner cortex does not contain X‐zone cells. It is interesting to note that *Frzb* is also expressed in the chicken adrenal cortex (Duprez et al. 1999), which features an intermingled structure of cortical and medullary cells. In chickens, it remains unclear whether the development of the functional cortex relies on the signaling interactions between cells in neighboring concentric cortical zones. Despite the presence of zF in both rats and mice, FRZB is expressed in zF in rats but not in mice. Instead, in mice, FRZB is specifically expressed in the inner cortex, with spatial and temporal expressions similar to many other X‐zone markers. This differential expression suggests the existence of species‐specific regulatory mechanisms that control the cellular expression of FRZB. Given that FRZB is a WNT inhibitor, the species‐specific differences in its cell‐type‐dependent expression raise important questions about the regulation and specificity of WNT signaling across species. Investigating the regulatory elements and transcriptional control of *Frzb* in different species could open new avenues for investigating the role of WNT in adrenal gland development. The species‐specific differences also shed light on the adrenal gland's evolutionary and functional adaptations and would help uncover the mechanisms driving WNT's cell‐type‐specific function. Moreover, in many tissues, WNT interplays with many other signaling pathways, including Hedgehog (Straube et al. 2025), androgen receptor (Lyraki et al. 2023), and TGF‐β pathways (Gumede et al. 2024), which also play roles in adrenal development (Penny et al. 2017). FRZB's function in the adrenal cortex may extend beyond WNT signaling modulation, potentially involving crosstalk with these pathways.

In summary, this comprehensive omics dataset provides a detailed view of transcriptomic changes in the adrenal gland throughout postnatal development, spanning from P0 to P49 in C57BL/6 mice, which roughly corresponds to human adolescence (up to ~18 years of age). The findings highlight dynamic, age‐ and sex‐dependent developmental processes and offer valuable opportunities to identify novel marker genes for specific adrenal cell types, as well as key genes and pathways involved in adrenal maturation. Importantly, the dataset uncovers transcriptomic patterns that extend beyond adrenal biology and contribute to our broader understanding of aging. For instance, the gradual decline in the expression of collagen‐related genes over time mirrors known age‐associated reductions in extracellular matrix components observed in other tissues, such as the skin. Such patterns underscore the potential of this dataset to inform aging research in multiple biological systems. While this study does not extend into late adulthood, where senescence and aging further impact adrenal function, it lays a critical foundation for future investigations into later‐life changes.

Together, these data serve as a valuable resource not only for adrenal researchers but also for the wider aging research community by enabling the exploration of a sex‐dependent gene expression dynamics that may underlie common aging‐related processes across tissues.

## Author Contributions

Y.K. and H.S.Z. collected the samples and performed RNA extraction. H.W., B.M., and C.‐C.J.H. analyzed data. Y.K. performed the immunostaining. H.W. and C.‐C.J.H. were major contributors in writing the manuscript. All authors read and approved the final manuscript.

## Conflicts of Interest

The authors declare no conflicts of interest.

## Data Availability

The datasets generated and/or analyzed during the current study are available in FigShare: (1) Lists of DEGs for Figures 1A,B and 4: 10.6084/m9.figshare.28038419. (2) The read count file for each sample: 10.6084/m9.figshare.28038422. (3) Gene lists of 24 WGCNA modules in Figure 5: 10.6084/m9.figshare.28038407 (the above data and materials have been deposited to FigShare and will be made available once the manuscript is accepted for publication).
